# Immobilization of β-Galactosidase onto Functionalized Graphene Nano-sheets Using Response Surface Methodology and Its Analytical Applications

**DOI:** 10.1371/journal.pone.0040708

**Published:** 2012-07-18

**Authors:** Devesh Kishore, Mahe Talat, Onkar Nath Srivastava, Arvind M. Kayastha

**Affiliations:** 1 School of Biotechnology, Faculty of Science, Banaras Hindu University, Varanasi, India; 2 Nanoscience and Nanotechnology Unit, Department of Physics, Banaras Hindu University, Varanasi, India; Argonne National Laboratory, United States of America

## Abstract

**Background:**

β-Galactosidase is a vital enzyme with diverse application in molecular biology and industries. It was covalently attached onto functionalized graphene nano-sheets for various analytical applications based on lactose reduction.

**Methodology/Principal Findings:**

Response surface methodology based on Box-Behnken design of experiment was used for determination of optimal immobilization conditions, which resulted in 84.2% immobilization efficiency. Native and immobilized functionalized graphene was characterized with the help of transmission and scanning electron microscopy, followed by Fourier transform infrared (FTIR) spectroscopy. Functionalized graphene sheets decorated with islands of immobilized enzyme were evidently visualized under both transmission and scanning electron microscopy after immobilization. FTIR spectra provided insight on various chemical interactions and bonding, involved during and after immobilization. Optimum temperature and energy of activation (*E_a_*) remains unchanged whereas optimum pH and *K_m_* were changed after immobilization. Increased thermal stability of enzyme was observed after conjugating the enzyme with functionalized graphene.

**Significance:**

Immobilized β-galactosidase showed excellent reusability with a retention of more than 92% enzymatic activity after 10 reuses and an ideal performance at broad ranges of industrial environment.

## Introduction

β-Galactosidase is an enzyme of industrial importance and has two main commercial applications in food technology; the reduction of lactose in dairy commodities for safe consumption by lactose intolerant peoples and production of galacto-oligosaccharides (GOS) via transgalactosylation reaction for a balanced gastrointestinal flora preservation.

Lactose, an integral component of breast milk, causes a discomfort in most of the children and adolescent peoples worldwide, in terms of abdominal pain, nausea, flatulence and bloating. The condition becomes more severe with advancement of age due to dropping in the gastric β-galactosidase secretion. Now a days, consumers are becoming more and more conscious regarding influence of diet on health and demanding natural foods with beneficial health effects and luscious taste. Therefore, commercially reduced lactose products are being manufactured for peoples across the globe.

Galacto-oligosaccharides (GOS) are non-digestible sugars containing two to five molecules of galactose and one molecule of glucose or lactose connected through glycosidic bonds. In general, transgalactosylation dominates early in the reaction, producing GOS with a high yield. As lactose conversion increases, the enzymatic hydrolysis activity takes over transgalactosylation; resulting complete conversion of lactose into glucose and galactose units. GOS can be classified as prebiotics or ‘Bifidus growth factor’ because of their selective stimulation of bifidobacteria in the lower intestine [1]. Health benefit offers by GOS includes reduction of detrimental bacteria [1], [2], promoting of vitamin producing flora [3], prevention of constipation and increase in mineral absorption [4], GOS have shown to be very stable at high temperatures and low pH. Therefore, GOS can also be used in a variety of products, including fermented milk products, breads, jams, confectionery, beverages, etc [5].

Enzymes are too expensive to be discarded after single use, which makes their commercial exploitation uneconomical. This obstruction can be addressed by coupling of enzyme with a suitable support material. Compared with soluble β-galactosidase, immobilized β-galactosidase may provide many advantages in production of lactose reduced dairy commodities, such as high enzyme reusability, high yield, improvement of thermal stability, continuous operation, controlled product formation, high reactor productivity, no contamination of product by the enzyme with simplified and efficient processing.

The composition, morphology, and surface characteristics of support material also play critical roles in regulating the activity of immobilized enzyme. Ideally, the supporting material should prevent enzyme aggregation or denaturation, but would maintain the native conformation of the enzymes [6]. A large number of support materials have been used for enzyme attachment such as affinity matrix [7], glass [8], magnetic beads [9], nano-materials [6], [10], Amberlite and Chitosan [11]. Nano-materials always have advantages and preferences over bulk materials in terms of their miniature size, large surface area with high enzyme loading capacity and aqueous suspendability for uniform distribution throughout reaction mixture. Therefore, nano-materials are getting more attentions for utilization as a support material for efficient enzyme immobilization.

Graphene has recently emerged as a new fascinating carbon based nano-material of applications, because of its unique mechanical and electronic properties [12]. It is a counterpart of graphite with well separated single layers of carbon packed in a hexagonal (honeycomb) lattice. Graphene, unlike other regular nano-particals, provide single carbon atoms thick graphite sheets with enormous surface area; perfect for uniform attachment of biomolecules. In addition, being made of carbon atoms, it does not alter native biochemical properties of attached biomolecules significantly. Graphene based material has recently been used in a variety of technical applications such as biofuel cells, biosensing material, drug delivery and catalysis [13], [14], [15], [16]. Biocompatibility issues of graphene oxide on animal models have also been studied and demonstrated non-toxic effects of the material under low dose administration [14], [17], [18], [19], [20].

Optimization of biochemical coupling can be done by employing either univariate or multivariate strategies. Univariate procedure may fail since the effect of one variable may be dependent on the level of others involved in the optimization. Multivariate optimization schemes involve experimental designs for which the levels of all the variables are changed simultaneously. Response surface methodology (RSM) has become very popular in recent years, with wide range of applications in biochemical process optimization [8], [21], [22], [23]. It offers advantages such as considerable reduction in the number of experiments to be executed, results in less reagents consumption and less laboratory work. Furthermore, this method allows the development of mathematical models that permits assessment of the relevance, statistical significance of the factors being studied as well as evaluates the interaction effects between the factors [24].

**Figure 1:**
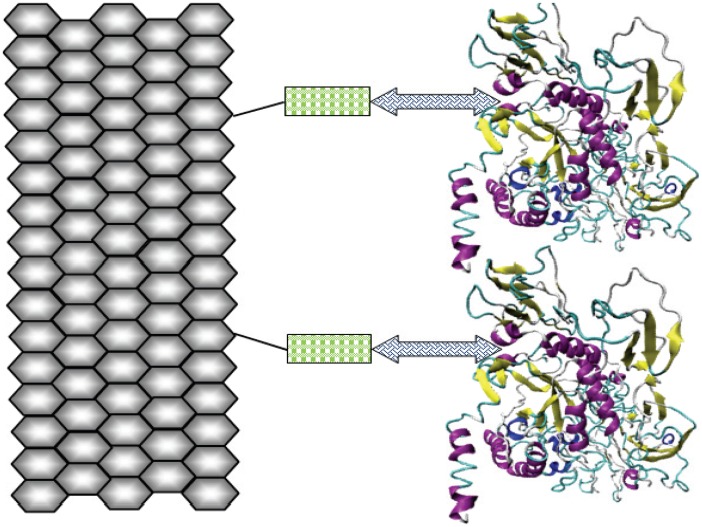
Schematic representation. Functionalized graphene nano-sheet (hexagonal arrangement) binds to the thiol group of cysteamine monolayer (rectangles). Glutaraldehyde cross-linker (double-headed arrows) provide binding terminus for both cysteamine monolayer and β-galactosidase.

β-Galactosidase is a ubiquitous enzyme and has been isolated from several organism. In addition, many micro-organisms produce significant amount of β-galactosidase including *Kluveromyces lactis* [25], *Escherichia coli* [26], *Bulleria singularis* [27], *Thermotoga maritime* [28], *Lactobacillus reuteri* [29], among others. β-Galactosidase could be isolated from these organisms and employed for hydrolysis of lactose contents of dairy commodities. However, it cannot be accepted from all organism, especially, when the enzyme is to be used in food systems, due to its “generally regarded as safe” (GRAS) status or due to commercial scarcity or unavailability in sufficient amount for industrial applications. Therefore, a β-galactosidase from plant source (*Cicer arietinum*) has been isolated and used for covalent attachment to functionalized graphene using RSM (Fig. 1). The enzyme kinetics has been also studied to obtain best catalytic performance and enhanced reusability with greater storage ability. In addition, the attachment was characterized by means of Scanning and Transmission Electron Microscopy (SEM, TEM), followed by Fourier transform infrared (FTIR) analysis. Its exploitation can be achieved in production of lactose reduced dairy commodities and galacto-oligosaccharides at industrial scale, which could be used for harmless consumption by lactose intolerant individuals.

## Results

### Process Optimization via RSM

Chick pea β-galactosidase (*Cp*GAL) was extracted and purified from chick pea seeds by employing various chromatographic techniques as described recently [30]. Finally purified enzyme showed a specific activity of 220 (±15) Units mg^−1^ and was found to be homogeneous when ran on SDS-PAGE.

**Figure 2:**
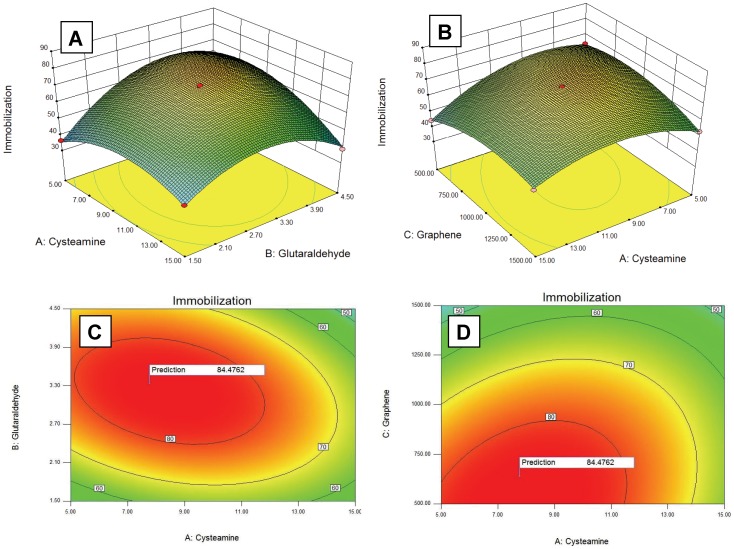
Response surface plots showing effects of various parameters on % immobilization efficiency (A–B) and contour plots showing predicted optimal response (C–D).

Response surface methodology was employed for getting optimal conditions for maximal enzyme immobilization. RSM mainly exploits two designs of experiments for process optimization viz., Central Composite design and Box-Behnken design. Ferreira et al., [24] made a comparative study between these two designs of experiment and concluded that Box-Behnken design is more efficient than Central Composite design. Therefore, in the present study, we used Box-Behnken design of experiment for variables optimization. In the preliminary work, the effect of the independent parameters on response was investigated by varying one factor at a time; to select operational ranges of variables where response (immobilization) was most favourable (data not shown). Subsequently RSM was applied within these ranges of parameters to obtain maximum response. Detailed experimental results are presented in Table 1 as actual immobilization percentage (±0.5). Based on these experiments, a mathematical model was build to obtain the maximum immobilization response within the set range of variables and following points were determined (Fig. 2):

**Table 1:** Box-Behnken experimental design for independent variables and their corresponding observed and predicted values of response.

Run	Cysteamine (mM)	Glutaraldehyde (% v/v)	FunctionalizedGraphene (µg)	Enzyme (µg)	Immobilization (%)	
					Actual	Predicted
**1**	5	3	500	600	64.2	63.74
**2**	15	4.5	1000	600	38.4	39.71
**3**	10	3	1500	900	56.3	56.4
**4**	10	1.5	500	600	44.2	44.37
**5**	15	1.5	1000	600	41.5	41.15
**6**	10	1.5	1000	300	30.2	30.66
**7**	15	3	1000	900	65.3	64.08
**8**	10	1.5	1000	900	56.4	56.94
**9**	10	3	1000	600	72.5	72.76
**10**	10	1.5	1500	600	40.1	40.28
**11**	10	3	1500	300	63.2	62.86
**12**	15	3	500	600	44.4	44.61
**13**	10	3	1000	600	73.5	72.76
**14**	10	3	500	900	83.5	84.13
**15**	10	3	1000	600	73.1	72.76
**16**	5	3	1000	900	68.2	68.32
**17**	15	3	1000	300	39.1	38.4
**18**	5	4.5	1000	600	62.3	62.95
**19**	15	3	1500	600	54.2	54.92
**20**	10	4.5	1500	600	58.1	57.35
**21**	5	3	1500	600	53.2	53.26
**22**	10	4.5	1000	900	65.1	64.91
**23**	5	1.5	1000	600	36.4	35.38
**24**	10	4.5	500	600	54.2	53.43
**25**	10	3	1000	600	72.1	72.76
**26**	10	4.5	1000	300	49.1	48.83
**27**	10	3	500	300	35.1	35.3
**28**	10	3	1000	600	72.6	72.76
**29**	5	3	1000	300	51	51.63

Cysteamine: 7.77 mM; Glutaraldehyde: 3.34% (v/v); Functionalized graphene: 640.06 µg; Enzyme: 896.14 µg; Immobilization: 84.47%.

Supplementary experiments were carried out for validation of these predicted values and about 84.20% immobilization was achieved, which was in agreement with the predicted value. Multiple regression analysis was applied to establish the polynomial coefficients and a relationship between immobilization percentage and independent variables was established by means of a quadratic polynomial equation. The final equation to determine the immobilization efficiency can be summarized as follows:
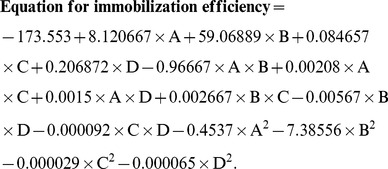
where A, cysteamine concentration (mM); B, glutaraldehyde concentration (%; v/v); C, amount of functionalized graphene (µg) and D represents amount of enzyme (µg).

Analysis of variance (ANOVA) was performed to establish the adequacy and significance of predicted quadratic model as given in Table 2. The Model F-value of 550.96 implies significance of the model. The “Lack of Fit F-value” of 2.97 implies the Lack of Fit is good and not significant relative to the pure error. The “Pred R-Squared” of 0.9905 is in reasonable agreement with the “Adj R-Squared” of 0.9964. A ratio greater than 4 was desired for “Adeq Precision” which measures the signal to noise ratio. The ratio was found to be 87.746 that indicates an adequate signal.

**Table 2:** Analysis of variance (ANOVA) for response surface model.

Source	Sum of Squares	Df	Mean Square	F-Value	p-value (Prob > F)	
**Model**	5537.76	14	395.55	550.96	<0.0001	significant
**A-Cysteamine**	228.81	1	228.81	318.7	<0.0001	
**B-Glutaraldehyde**	512.21	1	512.21	713.45	<0.0001	
**C- Functionalized graphene**	0.02	1	0.02	0.03	0.8672	
**D-Enzyme**	1346.2	1	1346.2	1875.09	<0.0001	
**AB**	210.25	1	210.25	292.85	<0.0001	
**AC**	108.16	1	108.16	150.65	<0.0001	
**AD**	20.25	1	20.25	28.2	0.0001	
**BC**	16	1	16	22.29	0.0003	
**BD**	26.01	1	26.01	36.23	<0.0001	
**CD**	764.52	1	764.52	1064.89	<0.0001	
**A^2^**	834.5	1	834.5	1162.36	<0.0001	
**B^2^**	1791.19	1	1791.19	2494.9	<0.0001	
**C^2^**	343.77	1	343.77	478.83	<0.0001	
**D^2^**	218.58	1	218.58	304.46	<0.0001	
**Residual**	10.05	14	0.72			
**Lack of Fit**	8.86	10	0.89	2.97	0.1526	not significant
**Pure Error**	1.19	4	0.3			
**Cor Total**	5547.81	28				

R-Squared = 0.998188; Adj R-Squared = 0.996377; Pred R-Squared = 0.990466.

### Characterization

The functionalized graphene used in the present investigation was prepared by thermal exfoliation of graphite oxide following the method given by Staudenmaier’s method [31], [32]. Exfoliation of the stacked structure occurs through the extrusion of carbon dioxide generated by heating graphite oxide to 1050°C. The high temperature gas creates enormous pressure within the stacked layer [33]. The size of starting graphite flakes doesn’t affect the size of produced functionalized graphene, suggesting that the product size is intrinsically determined by the oxidation process. Prepared functionalized graphene sheets did not collapse back to graphite oxide due to the wrinkled nature, but were highly agglomerated. After dispersion by ultrasonication in appropriate solvents, analysis by atomic force microscopy and statistical analysis has shown that 80% of the observed flakes were single sheets [32], [33].

**Figure 3:**
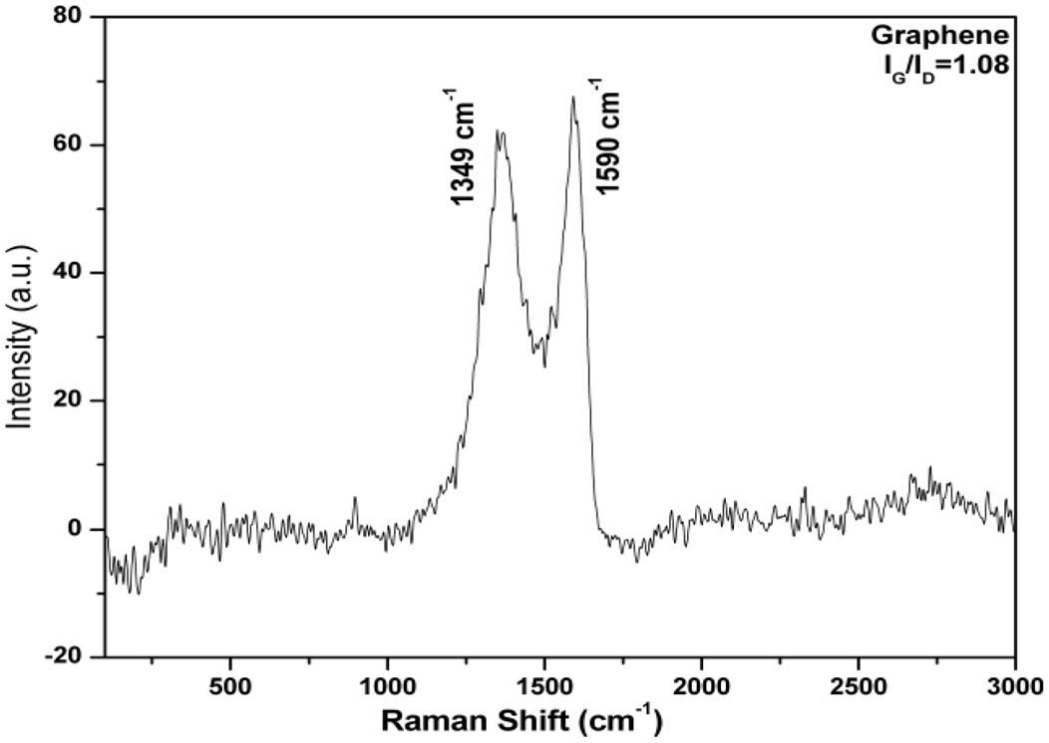
Raman spectra of functionalized graphene.

Prepared functionalized graphene was characterized using Raman spectroscopy. The stretching of the C-C bond in graphitic materials gives rise to the so called G-band Raman feature, which is common to all sp^2^ carbon systems and the presence of certain amount of disorder or edges within the structure results into a D-band. The characteristic peak at 1349 cm^−1^ of D peak (associated with the order/disorder of the system) and at 1590 cm^−1^ of G peak (an indicator of the stacking structure) confirmed the formation of functionalized graphene (Fig. 3). The I_G_/I_D_ ratio was used as a mean of determining the number of layers in a graphene sample and its overall stacking behaviour; where high ratio indicated high degree of exfoliation. In the present case, the ratio was found to be 1.08.

**Figure 4:**
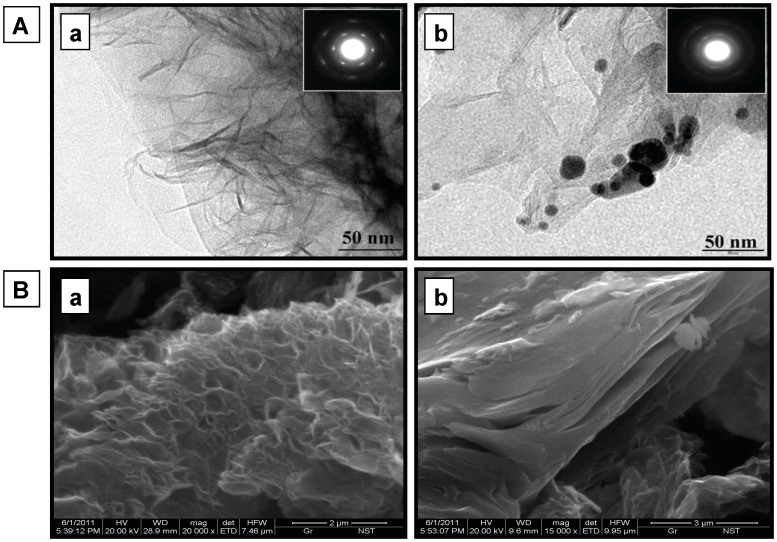
Micrographs of functionalized and immobilized graphene nano sheets. (**A**) Transmission Electron Micrograph (TEM) images of functionalized (**a**) and coupled (**b**) graphene showing fine transparent sheets with inset showing the characteristic Selected Area electron Diffraction pattern (SAD). Functionalized graphene sheets appear transparent whereas islands of immobilized enzyme can be seen in coupled graphene (**B**) Scanning Electron Micrograph (SEM) images of functionalized (**a**) and coupled (**b**) graphene.

Functionalized graphene was systematically characterized using SEM and TEM, followed by FTIR spectroscopy. Figure 4 shows bright field TEM images, substantiating typical transparent sheets of functionalized graphene at 50 nm resolution with characteristic Selected Area electron Diffraction (SAD) pattern in the inset. Islands of immobilized enzyme on the functionalized graphene sheets can be evidently seen in the TEM images, which were absent in the functionalized graphene TEM images. The immobilization also leads to an alteration in characteristic SAD pattern of functionalized graphene (inset). Attachment of enzyme could also be seen distinctly through SEM images, which present visual evidences in support of TEM images.

**Figure 5:**
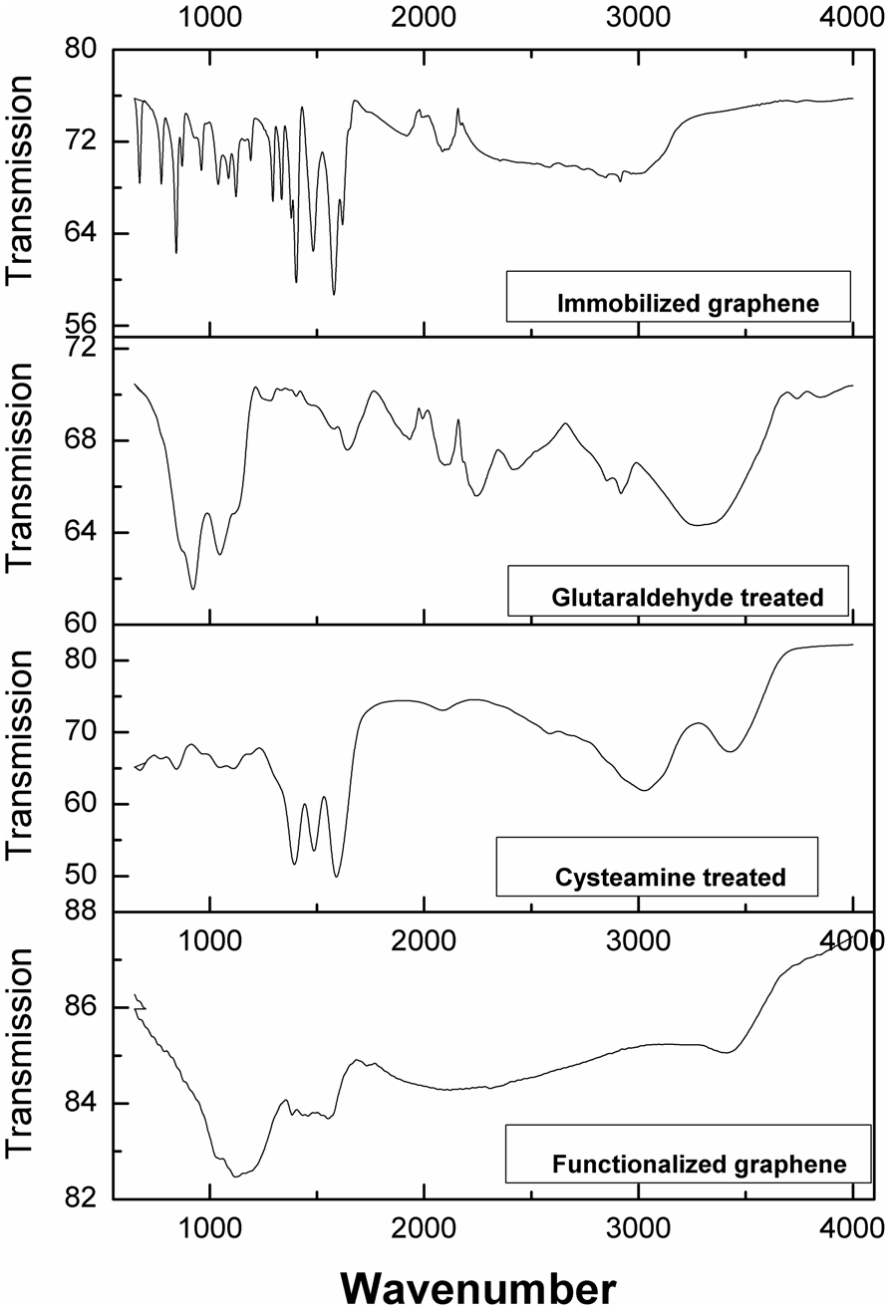
FTIR spectra of immobilized, glutaraldehyde treated, cysteamine treated and functionalized graphene respectively.

FTIR spectra were taken for native, cysteamine treated, glutaraldehyde treated and enzyme immobilized graphene sheets for getting an inside regarding interaction between functionalized graphene and enzyme molecules (Fig. 5). A drastic difference was observed in four FTIR spectra. A description of vibration peaks corresponding to functionalized graphene and their assignment to the respective functional class at different stages of immobilization is given in Table 3. Immobilized graphene exhibits many prominent bands in the region of 675 to 1350 cm^−1^, which can be attributed to aliphatic and aromatic amine groups of attached enzyme The complexity of infrared spectra in the 1450 to 600 cm^−1^ region makes it difficult to assign all absorption bands, and because of the unique patterns found there, it is often called the fingerprint region whereas spectra in 4000 to 1450 cm^−1^ region is usually due to stretching vibrations of diatomic units, and called the group frequency region.

**Table 3:** FTIR vibration peaks and their corresponding functional group.

Vibration assignment	Functionalized graphene	Cysteamine treated graphene	Glutaraldehydetreated graphene	Enzyme coupled graphene
**C-H deformation**				673
**C-H bending of arenes**		847	864	775, 844, 872
**N-O aliphatic**		971	923	962
**C-O stretch**	1030, 1120	1112	1131	1124
**C = S stretch**		1049	1048	1040, 1089
**C = O amide I bond**				1192, 1296
**O-H bending in plane**	1385	1396		1336, 1382, 1403
**Amide II bond**		1487, 1592		1481, 1576
**Amide I band**			1643	1621
**C = C asymmetric stretch**			1934	1908
**-N = C = O or C?C stretch**		2088	2097, 2244	2099
**O-H (acids) stretch**		3028	2418, 2919	2916
**O-H stretch**	3436	3426	3428	

FTIR of functionalized graphene showed bands of carbonyl stretch (C = O) of carboxylic group at 1030 cm^−1^. Peaks at 1385 and 1120 cm^−1^ may be attributed to C-OH (hydroxyl) and C-O (epoxy), respectively; therefore confirming the presence of oxygen containing functional groups e.g. C = O, C-OH and C-O on functionalized graphene sheets. Cysteine functionalizes graphene sheets through –SH group as evident from thicarbonyl (C = S) stretch at 1049 cm^−1^, while other end containing –NH_2_ group remains available for glutaraldehyde attachment. Treatment with cysteine also contributes amide II peaks at 1487 and 1592 cm^−1^ and free hydroxyl stretch peak at 3028 cm^−1^. Next, the functionalized graphene sheets were treated with glutaraldehyde solution. One arm of glutaraldehyde binds to the -NH_2_ group of cysteamine through –CHO group while the other arm remains free for attachment with enzyme via lysine residues. FTIR at this step showed bands corresponding to –N = C = O stretch at 2097 and 2244 cm^−1^. Final step involves attachment of enzyme to the free arm of glutaraldehyde via lysine amino group. Resultant FTIR spectra showed many prominent peaks in the region of 673–1576 cm^−1^, which can be correlated to various bonds and stretch as stated in Table 3. Bands at 1192 and 1296 cm^−1^ represent carbonyl amide I bonds whereas bands at 1481 and 1567 cm^−1^ represents amide II bonds.

UV-Vis spectra were also recorded for functionalized graphene sheets, before and after immobilization (Fig. S1). Functionalized graphene did not show any peak in UV-Vis; whereas immobilized graphene prominently absorbs around 280 nm due to the attached enzyme.

SEM and TEM images altogether with enzyme activity assays, FTIR and UV-Vis spectra manifest covalent attachment of enzyme onto functionalized graphene sheets.

### Steady State Kinetics

Enzyme immobilization often brings about an alteration in kinetics parameters [11], [34]. Therefore, kinetics parameters were estimated for a second time to obtain the optimal performance of attached enzyme. Immobilized enzyme showed an optimum pH of 3.8 with ONPG, whereas it was found to be 2.8 for soluble enzyme. Optimum pH with lactose was 4.0 for soluble enzyme and remains unchanged after immobilization.

**Figure 6:**
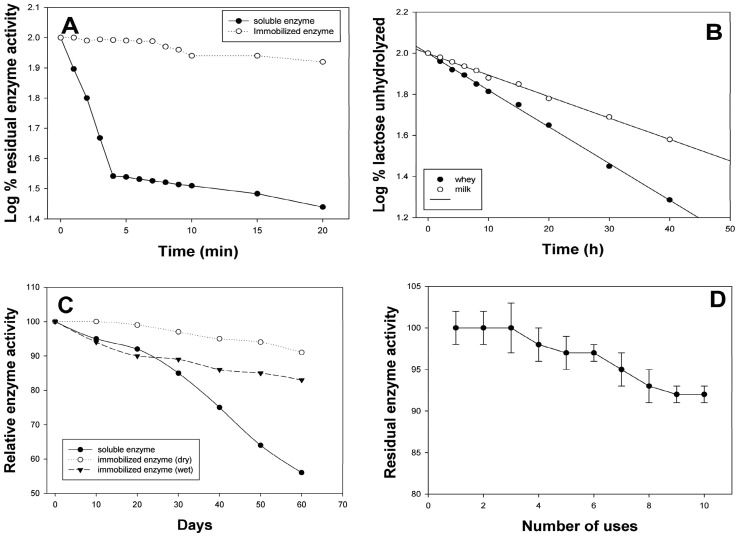
Stability, kinetics and reusability of immobilized enzyme. (**A**) Represents thermal stability; (**B**) represents enzymatic activity of immobilized *Cp*GAL against lactose hydrolysis with respect to time. (**C**) **&** (**D**) Represents storage stability at room temperature and reusability of immobilized *Cp*GAL, respectively (see material and methods for further detail).

Similar to *A. oryzae* β-galactosidase [35], *Cp*GAL did not exhibit any change in optimum temperature after immobilization and remains 60°C for both soluble and immobilized enzyme, when assayed with lactose under standard condition. However, thermal stability was significantly improved after immobilization. The immobilized enzyme could be kept at 60°C for 10 min without any significant loss in enzymatic activity (Fig. 6A) whereas soluble enzyme loses 64% residual activity within 4 min at the same temperature [8]. A small difference in the *K_m_* value was also observed after immobilization of enzyme as it changed from 1.73 mM to 1.28 mM with ONPG and 10 mM to 5.78 mM with lactose. Galactose was found to be competitive inhibitor for both free and immobilized enzyme; however *K_i_* value increased from 2.44 to 3.89 mM, respectively. Glucose does not significantly affect bioconversion abilities of both soluble and immobilized enzyme up to 50 mM concentration. Energy of activation (*E_a_*) for lactose (10.50±0.2 kcal mol^−1^) did not change after immobilization. Table 4 summarize a comparative account of optimal enzyme kinetics, before and after immobilization. Similar changes in kinetic parameters have been observed for various enzymes after immobilization [8], [10], [11], [34].

**Table 4:** Kinetic parameters of soluble and immobilized enzyme.

	Soluble		Immobilized	
	ONPG	Lactose	ONPG	Lactose
**Optimum pH**	2.8	4.0	3.8	4.0
**Optimum** **temperature**	60°C	60°C	60°C	60°C
**K_m_**	1.73 mM	10 mM	1.28 mM	5.78 mM
**K_i_**	–	2.44 mM	–	3.89 mM

Diffusion coefficient also plays a key role in alteration of steady state kinetics of an immobilized enzyme, which in turn depends upon the size, charge and enzyme loading capacity of support material. The overall bioconversion rate is governed by liquid film mass transfer or external diffusion as internal diffusion does not exist in a non-porous support material. External diffusion can be defined in term of Damköhler number (Da), a dimensionless ratio of reaction velocity to mass transfer velocity as
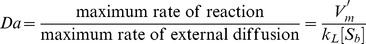
where 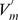 is the maximum reaction rate per unit of external surface area, *k*_L_ is the liquid mass transfer coefficient and S_b_ is the substrate concentration in bulk solution. When Da >>1, the external diffusion rate becomes limiting. Liquid mass transfer coefficient (*k*_L_) depends upon the diffusivity of the substrate and the effective distances between the surface and the bulk phase [36] and can be defined as



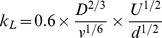
where D is mass diffusivity of the substrate in the liquid phase (a function of temperature and pressure), ν is the kinematic viscosity, U is the free-system liquid velocity of the fluid flowing past the particle and d is the size of immobilized particle. The Damköhler number is inversely proportional to the size of immobilized particle and can be increased by reducing the size of immobilization matrix, which results in higher catalytical abilities and bioconversion rates of an enzyme. Diffusion and mass transfer limitation of immobilized β-galactosidase has been encountered by various authors in recent years [10], [37], [38].

### Lactose Hydrolysis

The functional aspects of enzyme, coupled with functionalized graphene nano-sheets were also determined by estimating its hydrolyzing capacities for milk and whey lactose in batch reactor mode at an analytical scale. Rate of lactose hydrolysis and t_1/2_ was determined as stated in methods and were found to be 0.0413 h^−1^ and 16.78 h for whey lactose hydrolysis. The values for milk lactose hydrolysis were found to be 0.0238 h^−1^ and 29.34 h, respectively (Fig. 6B). Whey lactose was preferably hydrolysed over milk lactose because of its comparative acidic pH, where optimal bioconversion abilities of immobilized enzyme approaches. Similar pattern of whey and milk lactose hydrolysis has been observed for other immobilized β-galactosidase. Pea β-galactosidase immobilized onto Sephadex and Chitosan exhibited milk lactose hydrolysis at room temperature with t_1/2_ values to be 99.97 and 5.17 h, respectively; whereas these values for whey lactose hydrolysis were reported to be 33.36 and 4.61 h, respectively [38].

### Storage Stability and Reusability

Excellent storage ability without losing substantial biocatalytical efficiency is the key factor for any industrially important enzyme and often influences the final assessment over other available enzymes in the similar domain. Storage stability is an intrinsic property of the enzyme and varies with different sources of extraction; however it can be improved to a great extent by cross-linking the enzyme with a suitable matrix [8], [10], [38]. The attachment seizes most of the intramolecular undulations and gives an internal strength to enzyme, essential for preservation of proper backbone structure in three dimensions.

To study the storage stability, the immobilized enzyme was divided into aliquots and stored in wet (25 mM sodium phosphate buffer, pH 6.0) and dried conditions at 4°C and room temperature (27–30°C). The enzymatic activity was calculated every week. The enzyme was found to be moderately stable under all tested conditions. Immobilized enzyme could retain more than 94% residual activity for 4 months in both dry and wet conditions, when stored at 4°C. At room temperature, the immobilized enzyme preserved 91% residual activity when stored in dry conditions, whereas samples stored in wet conditions exhibit 83% residual activity after 2 months (Fig. 6C).

Reusability of a biocatalyst is another important factor that considerably reduces the processing cost of any industrial merchandise. Purification of enzyme is a tedious process; involving expensive resources, enormous laboratory work and often end up with very low yield (generally ≥15%). There are many reports which show improved stability of the enzymes through supported attachment via covalent bonding [10], [11]. Immobilized enzyme with good reusability and prolonged stability provides an economical gain and can be used constantly in both batch and continuous reactors.

Functionalized graphene coupled enzyme stored at 4°C showed excellent reusability with negligible loss up to three cycles and a retention of more than 92% enzymatic activity after 10 cycles of repeated use (Fig. 6D). Improvement in stability and reusability of enzymes following immobilization has been well-documented [8], [10], [35], [38].

## Discussion

The activity of β-galactosidase tends to decrease markedly during immobilization due to damage at the active site and distortion of the native structure caused by covalent bonding between enzyme and support matrix. This damage could be reduced significantly by pre-treatment of enzyme with lactose, which shields the active site from deformation during immobilization process through steric effects [34]. Therefore, pre-treatment of *Cp*GAL with lactose was done before starting any immobilization experiments. Factors like enzyme carrier and chemical reactants related to the immobilization process also have to be considered during immobilization, so that low cost operation can be achieved in industrial processing. Chemisorptions of cysteamine monolayer was employed via its thiol group as a linkage layers for adsorption of glutaraldehyde. Glutaraldehyde was used as a cross-linker, which also offers additional advantages in view of its GRAS status, low cost, high efficiency, and stability [39].

In cheese industry, lactose is a waste, which causes several economical and environmental problems. Approximately 47% of the whey produced annually worldwide is disposed off [40]. Therefore, conversion of lactose into a highly valuable product such as GOS using continuous or batch reactor mode with immobilized enzyme system is of high interest to the food industry. Transgalactosylation reactions take place prominently at high concentration of lactose; which disturbs the balance of glucose and galactose in the reaction mixture. Glucose starts populating in the reaction mixture as galactose is continuously being consumed for GOS production. Eventually the concentration of glucose limits the inhibition of enzyme. Thus, an enzyme with significantly low glucose inhibition of lactose hydrolysis is obviously desirable [41]. *Cp*GAL does not exhibit significant glucose inhibition up to 50 mM concentration in reaction mixture; therefore could be best suitable for GOS production using transgalactosylation reactions.

Biosensor applications also require a highly active immobilized enzyme system that allows the maintenance of an efficient link between sensing molecule and a transduction component [37]. *Cp*GAL coupled to functionalized graphene with such high enzyme loading capacity and signal conductivity offers new opportunities to combine discrete bioconversion abilities and amplified sensing technology for fabrication of a lactose sensing device. Immobilized graphene can be coupled with some glucose sensing amperometric or clark electrode [42] for real-time detection of lactose in dairy based industries. High conductivity of graphene can also be exploited by fabricating a third generation oxygen sensing electrode by co-immobilization of *Cp*GAL with glucose oxidase. One such glucose sensing device has recently been formulated using glucose oxidase, covalently attached to functionalized graphene nanoplates [14]. Alternatively, immobilized graphene can also be used with a commercially available glucose sensing device. There are many rapid glucose detection kits available in the market. One of such kit has recently been used by Dwevedi et al., [10] to make a lactose sensing nanoprobe. These types of lactose sensing devices are in much demand and have promising applications in both clinical and industrial fields.

## Materials and Methods

Dry seeds of *Cicer arietinum* were purchased from local market. The chemicals for buffers preparation were of analytical or electrophoresis grade from Merck Eurolab GmbH Damstadt, Germany. All other chemicals and reagents were purchased from Sigma Chem. Co. Milli Q (Millipore, Bedford, MA, USA) water with a resistance of higher than 18 MΩ cm was used all throughout the experiments.

Enzyme was purified as stated previously [30]. Homogeneity of the purified preparation was checked by SDS-PAGE and size-exclusion chromatography. Stock solution of lactose (100 mM prepared in 25 mM phosphate buffer, pH 6.0) was added to the purified enzyme solution to make a final preparation containing 2 mg mL^−1^ enzyme in 20 mM lactose in the same buffer. This β-galactosidase solution was then used for immobilization as described in methods.

Activity of both free and immobilized enzyme towards *o*-nitrophenyl-β-D-galactopyranoside (ONPG) and lactose were estimated as given previously [8]. During optimization of *Cp*GAL immobilization onto functionalized graphene, activity was measured using ONPG as substrate due to its higher sensitivity than lactose. Immobilization efficiency was calculated as given below:


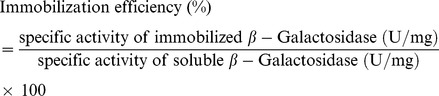


Protein estimation was done by using the Bradford method [43], using crystalline bovine serum albumin as standard protein.

### Functionalized Graphene Sheet Preparation and Characterization

Functionalized graphene was synthesized by thermal exfoliation of graphite oxide. Graphite powder (<50 µm, 1 g) was reacted with strong oxidizing solution of conc. H_2_SO_4_, HNO_3_ and potassium chlorate at room temperature with constant stirring condition. As obtained, graphite oxide solution was washed with distilled water and 10% HCl solution to remove sulphate and other ionic impurities followed by drying at 80°C under vacuum. Next, it was thermally exfoliated to synthesize graphene by rapid heating under an Ar atmosphere. Graphite oxide powder was placed in an alumina boat and inserted in to a 1.5 m long quartz tube with outer diameter of 25 mm. The sample was flushed with Ar gas for 15 min, and the quartz tube was quickly inserted in to a tube furnace pre-heated to 1050°C and held in the furnace for 30 sec. The functionalized graphene sample was cooled down to the room temperature under Ar gas flow. Prepared thermally exfoliated functionalized graphene differs from brownish graphite oxide as light weighted shiny black powder. Prepared functionalized graphene was suspended in double distilled water followed by sonication at room temperature for 10 min and left undisturbed for 30 min so that the larger non-exfoliated flakes settled down. Gently, suspended functionalized graphene was collected such that the larger flakes stay undisturbed.

The enzyme was coupled to the functionalized graphene with help of a spacer arm (cysteamine) and a cross-linker (glutaraldehyde). The functionalized graphene was dissolved in phosphate buffer (25 mM, pH 6.0) to make a final preparation containing 1 mg mL^−1^ graphene and divided in to 29 aliquots according to the design of experiment (Table 1). The graphene aliquots were equilibrated in the same buffer (1 mL reaction volume) for overnight followed by thorough rinsing with the same buffer. Next, it was treated with cysteamine, prepared in the same buffer (1 mL reaction volume) and kept under dark condition for 4 h at room temperature. After cysteamine treatment it was washed with the same buffer followed by glutaraldehyde treatment (1 mL reaction volume) under similar conditions for 4 h. Washing was done for one more time with phosphate buffer followed by incubation with the enzyme under dark conditions for 12 h at 4°C. The immobilized enzyme was washed thoroughly with chilled buffer (25 mM phosphate, ph 6.0) and immobilization was checked with activity assays under standard conditions. Each steps of immobilization was followed by thorough washing with chilled buffer (1 mL, washing two times) and centrifugation at 5000 rpm for 2 min at room temperature. Table 1 shows the list of independent variables according to the experimental design and their corresponding response.

Functionalized graphene sheets (both native and coupled) were characterized using transmission electron microscope (TEM; Technai 20 G^2^, 200 kV), scanning electron microscope (SEM; Philips: XL 20) and Fourier transform infrared spectroscopy (Perkin Elmer Spectrum 100 instrument). For TEM studies, a drop of sample was placed on electron microscope 200 mesh copper grid and allowed to evaporate the water to complete dryness of the sample, followed by loading into the machine. For SEM studies, samples were sprinkled on the stub having layer of silver glue for striking the particles. The fine structural details were obtained using secondary electron imaging mode. Fourier transform infrared spectroscopy was done in the range of 650 to 4000 cm^−1^ wave numbers, with 100 scans of each sample to obtain a good signal to noise ratio. Raman spectra were recorded using Raman spectrometer (Horiba Jobin Yvon HR-800) for vibrational characterization. Ar ion laser of 514.5 nm wavelength was used to excite the sample and the signal was collected in the backscattering geometry. UV-Vis spectra were recorded using NanoDrop (ND-1000, USA) Spectrophotometer.

### Experimental Setup and Statistical Analysis

A few preliminary experiments were carried out to determine the initial values of the factors, affecting the immobilization process (data not shown). Based on these experiments, the levels of the significant parameters and the interaction effects between various factors (amount of functionalized graphene, amount of enzyme and concentration of cysteamine and glutaraldehyde), which significantly influence the immobilization, were analysed and optimized using Box–Behnken design of experiment. In this study, the experimental plan consisted of 29 trials and the independent variables were studied at three different levels. The variables and their levels selected for obtaining immobilization of *Cp*GAL onto functionalized graphene were: amount of functionalized graphene (500, 1000, 1500 µg), cysteamine concentration (5, 10 and 15 mM), glutaraldehyde concentration (1.5, 3.5 and 4.5%; v/v) and amount of enzyme (300, 600 and 900 µg). All the experiments were done in duplicates and the average of immobilization obtained was taken as the dependent variable or response (Y). Experimental design and analysis was done using ‘Design Expert’ software (Version 8.0, Stat-Ease Inc., Minneapolis, USA). The mathematical relationship relating the variables to the responses can be calculated by the quadratic polynomial equation:
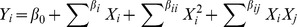
where *Y_i_* is the predicted response, *X_i_X_j_* are input variables which inuence the response variable *Y*; *β_0_* is the offset term; *β_i_* is the *i* ^th^ linear coefficient; *β_ii_* the *i* ^th^ quadratic coefficient and *β_ij_* is the *ij* ^th^ interaction coefficient. Statistical analysis of the model was performed to evaluate the analysis of variance (ANOVA) which included lack of fit, Fisher’s F-test, its associated probability p(F) and correlation coefficient R to measures the goodness of quadratic model. For the present study, a total of 29 tests were performed to estimate the coefficients. The generated mathematical model was validated by conducting experiment at given optimal conditions.

### Steady State Kinetics

The optimum pH for β-galactosidase (both free and immobilized) were studied with respect to ONPG and lactose in the pH range of 3.0–7.0 (Glycine-HCl: pH 3.0–4.0, Acetate buffer: 4.0–5.5, Sodium phosphate: 5.5–7.0). In all cases, 50 mM buffers were used and activity was observed under standard conditions. The optimum temperature was studied for both free and immobilized enzyme by assaying the enzyme activity with lactose and ONPG, at temperature between 25–75°C in their respective optimum pH buffers for 10 min. The temperature stability (thermal inactivation) was studied by incubating the free and immobilized enzyme at various temperatures for different time intervals, followed by residual activity assay under standard condition. Activation energy (E_a_) was calculated from the slope of the curve using Arrhenius plot. The enzyme activity as a function of substrate concentration was measured for both free and immobilized enzyme by varying substrate concentration using standard assay procedure. Data was plotted and analysed using Lineweaver-Burk plot for calculation of *K*_m_ and *V*_max_ with the help of Sigmaplot 11.0 version. Inhibition constant (*K*_i_) for galactose was determined using Dixon plot.

The aliquots of functionalized graphene coupled with enzyme were stored in both dry and wet (25 mM Sodium phosphate, pH 6.0) condition at 4°C and the residual activity was checked from time to time, with ONPG as substrate under given standard assay conditions. For reusability assessment, freshly immobilized enzyme was repeatedly used for 10 times and the residual activity was measured with ONPG as substrate. After each assay, the immobilized preparation was properly washed with 25 mM Sodium phosphate, pH 6.0; to remove any attached substrate that may obstruct during storage. Further, the immobilized β-galactosidase, which showed better stability, was reused over prolonged periods.

### Lactose Hydrolysis

Milk and whey lactose were prepared as stated previously [8]. The reaction was ignited by adding 1 mg enzyme linked functionalized graphene sheets in 5 mL of prepared milk or whey at room temperature (30±2°C). Aliquots of 20 µL were withdrawn at regular time interval and released glucose was estimated as described earlier in the methods. Percentage of unhydrolyzed lactose was calculated as:
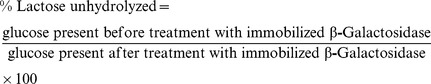
A plot was generated with log% lactose unhydrolyzed versus time, and rate constant of lactose hydrolysis was determined using the slope of the plot using the formula:



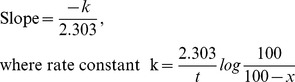
where ‘x’ is lactose unhydrolyzed. Therefore, time required for 50% lactose hydrolysis could be calculated as 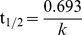.

## Supporting Information

Figure S1 **UV Vis spectra of functionalized and immobilized graphene nano sheets.**
(TIF)
