# Supplementary figures and images for: Immobilization of β-Galactosidase onto Functionalized Graphene Nano-sheets Using Response Surface Methodology and Its Analytical Applications
Source: PLoS One. 2012 Jul 18;7(7):e40708. doi: 10.1371/journal.pone.0040708 (PMC3399898; doi:10.1371/journal.pone.0040708)

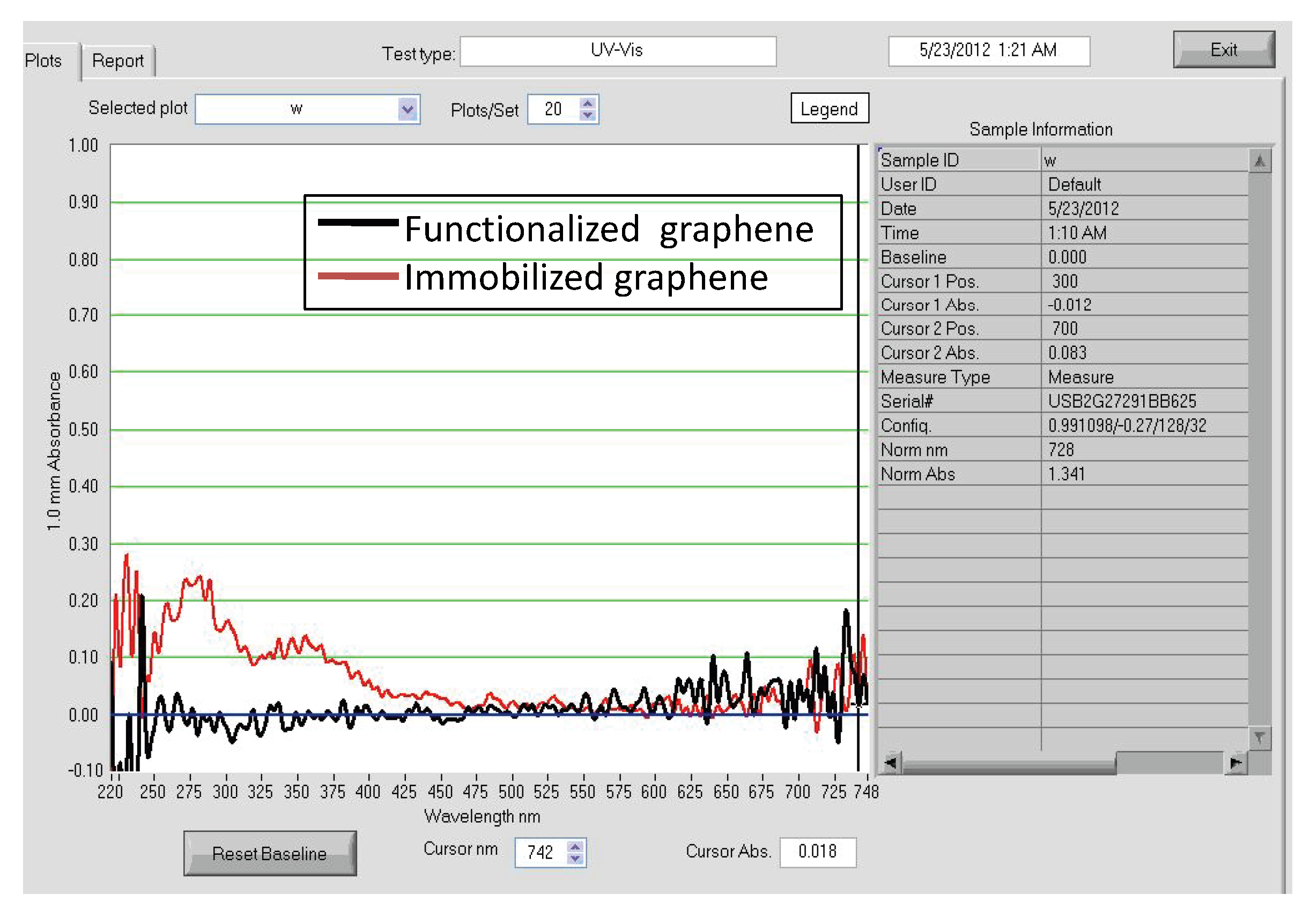

Supplement: Figure S1 — UV Vis spectra of functionalized and immobilized graphene nano sheets. (TIF) [file pone.0040708.s001.tif]
